# (*Z*)-1,3-Bis(4-chloro­phen­yl)-2-(1*H*-1,2,4-triazol-1-yl)prop-2-en-1-one

**DOI:** 10.1107/S1600536812016170

**Published:** 2012-04-21

**Authors:** Ling-Ling Dai, Ben-Tao Yin, Jing-Song Lv, Sheng-Feng Cui, Cheng-He Zhou

**Affiliations:** a Laboratory of Bioorganic & Medicinal Chemistry, School of Chemistry and Chemical Engineering, Southwest University, Chongqing 400715, People’s Republic of China

## Abstract

In the title mol­ecule, C_17_H_11_Cl_2_N_3_O, the C=C bond connecting the triazole and 4-chloro­phenyl groups adopts a *Z* geometry. The dihedral angles formed by the triazole ring and the 4-chloro substituted benzene rings are 67.3 (1) and 59.1 (1)°. The dihedral angle between the two benzene rings is 73.5 (1)°.

## Related literature

For the pharmacological activity of triazole compounds, see: Wang & Zhou (2011 ▶); Zhou & Wang (2012 ▶). For the biological activity of chalcones, see: Jin *et al.* (2010 ▶). For related structures, see: Wang *et al.* (2009 ▶); Yan *et al.* (2009 ▶). For the synthesis, see: Yin *et al.* (2012 ▶).
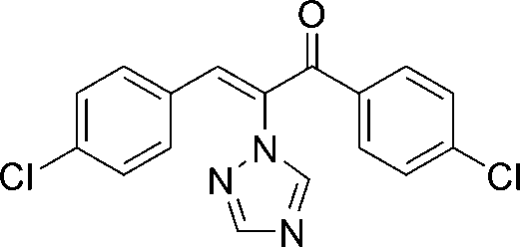


## Experimental

### 

#### Crystal data

C_17_H_11_Cl_2_N_3_O
*M* *_r_* = 344.19
Triclinic, 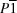

*a* = 5.588 (3) Å
*b* = 11.850 (7) Å
*c* = 12.653 (8) Å
α = 74.787 (10)°
β = 88.884 (9)°
γ = 86.461 (9)°
*V* = 807.1 (8) Å^3^
*Z* = 2
Mo *K*α radiation
μ = 0.41 mm^−1^
*T* = 296 K
0.22 × 0.18 × 0.15 mm

#### Data collection

Bruker APEXII CCD diffractometer
Absorption correction: multi-scan (*SADABS*; Bruker, 2009 ▶) *T*_min_ = 0.915, *T*_max_ = 0.941
4414 measured reflections
3104 independent reflections
2458 reflections with *I* > 2σ(*I*)
*R*_int_ = 0.013

#### Refinement

*R*[*F* ^2^ > 2σ(*F* ^2^)] = 0.037
*wR*(*F* ^2^) = 0.105
*S* = 1.02
3104 reflections
208 parameters
H-atom parameters constrained
Δρ_max_ = 0.21 e Å^−3^
Δρ_min_ = −0.26 e Å^−3^

### 

Data collection: *APEX2* (Bruker, 2009 ▶); cell refinement: *SAINT* (Bruker, 2009 ▶); data reduction: *SAINT*; program(s) used to solve structure: *SHELXS97* (Sheldrick, 2008 ▶); program(s) used to refine structure: *SHELXL97* (Sheldrick, 2008 ▶); molecular graphics: *SHELXTL* (Sheldrick, 2008 ▶); software used to prepare material for publication: *SHELXTL*.

## Supplementary Material

Crystal structure: contains datablock(s) global, I. DOI: 10.1107/S1600536812016170/lh5453sup1.cif

Structure factors: contains datablock(s) I. DOI: 10.1107/S1600536812016170/lh5453Isup2.hkl

Supplementary material file. DOI: 10.1107/S1600536812016170/lh5453Isup3.cml

Additional supplementary materials:  crystallographic information; 3D view; checkCIF report

## Figures and Tables

**Fig. 1.**
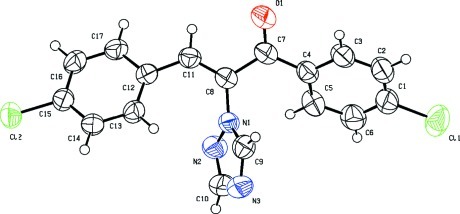
The molecular structure of (I), showing the displacement ellipsoids at the 50% probability level.

## supplementary crystallographic information

### Comment

Triazoles as an important type of five-membered aromatic heterocycles have been paid increasing attention for their broad bioactive spectrum in medicinal chemistry (Wang *et al.*, 2011; Zhou *et al.*, 2012). The incorporation of a triazole ring into chalcone skeletons could largely improve bioactivities like antimicrobial, anticancer, antiviral and anti-inflammatory (Jin *et al.*, 2010). In view of this, we have synthesized and reported some triazolylchalcones (Wang *et al.*, 2009; Yan *et al.*, 2009; Yin *et al.*, 2012). Herein, the crystal structure of title compound (I) is reported.

The molecular structure of (I) is shown in Fig. 1. The C8═C11 bond adopts a *Z* geometry. The atoms in the region of the double bond have an essentially planar arrangement *i.e.* the r.m.s. deviation the atoms C7/C8/C11/C12/N1 is 0.025 Å. The torsion angles of C12–C11═C8–C7 and C12–C11═C8–N1 are -174.66 (17)° and 5.7 (3)°. The dihedral angles formed by the triazole ring and the 4-chloro-substituted benzene rings are 67.3 (1)° (C1-C6) and 59.1 (1)° (C12-C17), respectively. The dihedral angle between the two benzene rings is 73.5 (1)°.

### Experimental

Compound (I) was prepared according to the procedure of Yin *et al.* (2012). Single crystals were grown by slow evaporation of a solution of (I) in ethyl acetate and petroleum ether (1:3, V/V) at room temperature.

### Refinement

H atoms were placed at calculated position with C—H = 0.93 Å. The *U*_iso_(H) values were set equal to 1.2U_eq_(C).

### Crystal data

**Table:**

C_17_H_11_Cl_2_N_3_O	*Z* = 2
*M**_r_* = 344.19	*F*(000) = 352
Triclinic, *P*1	*D*_x_ = 1.416 Mg m^−^^3^
Hall symbol: -P 1	Mo *K*α radiation, λ = 0.71073 Å
*a* = 5.588 (3) Å	Cell parameters from 2086 reflections
*b* = 11.850 (7) Å	θ = 3.3–27.2°
*c* = 12.653 (8) Å	µ = 0.41 mm^−^^1^
α = 74.787 (10)°	*T* = 296 K
β = 88.884 (9)°	Block, yellow
γ = 86.461 (9)°	0.22 × 0.18 × 0.15 mm
*V* = 807.1 (8) Å^3^	

### Data collection

**Table:**

Bruker APEXII CCD diffractometer	3104 independent reflections
Radiation source: fine-focus sealed tube	2458 reflections with *I* > 2σ(*I*)
Graphite monochromator	*R*_int_ = 0.013
φ and ω scans	θ_max_ = 26.0°, θ_min_ = 3.4°
Absorption correction: multi-scan (*SADABS*; Bruker, 2009)	*h* = −6→6
*T*_min_ = 0.915, *T*_max_ = 0.941	*k* = −7→14
4414 measured reflections	*l* = −14→15

### Refinement

**Table:**

Refinement on *F*^2^	Primary atom site location: structure-invariant direct methods
Least-squares matrix: full	Secondary atom site location: difference Fourier map
*R*[*F*^2^ > 2σ(*F*^2^)] = 0.037	Hydrogen site location: inferred from neighbouring sites
*wR*(*F*^2^) = 0.105	H-atom parameters constrained
*S* = 1.02	*w* = 1/[σ^2^(*F*_o_^2^) + (0.0515*P*)^2^ + 0.1439*P*] where *P* = (*F*_o_^2^ + 2*F*_c_^2^)/3
3104 reflections	(Δ/σ)_max_ = 0.001
208 parameters	Δρ_max_ = 0.21 e Å^−^^3^
0 restraints	Δρ_min_ = −0.26 e Å^−^^3^

### Special details

**Table:**

Geometry. All e.s.d.'s (except the e.s.d. in the dihedral angle between two l.s. planes) are estimated using the full covariance matrix. The cell e.s.d.'s are taken into account individually in the estimation of e.s.d.'s in distances, angles and torsion angles; correlations between e.s.d.'s in cell parameters are only used when they are defined by crystal symmetry. An approximate (isotropic) treatment of cell e.s.d.'s is used for estimating e.s.d.'s involving l.s. planes.
Refinement. Refinement of *F*^2^ against ALL reflections. The weighted *R*-factor *wR* and goodness of fit *S* are based on *F*^2^, conventional *R*-factors *R* are based on *F*, with *F* set to zero for negative *F*^2^. The threshold expression of *F*^2^ > σ(*F*^2^) is used only for calculating *R*-factors(gt) *etc*. and is not relevant to the choice of reflections for refinement. *R*-factors based on *F*^2^ are statistically about twice as large as those based on *F*, and *R*- factors based on ALL data will be even larger.

### Fractional atomic coordinates and isotropic or equivalent isotropic displacement parameters (Å^2^)

**Table:**

	*x*	*y*	*z*	*U*_iso_*/*U*_eq_	
Cl1	1.58438 (14)	0.60011 (6)	−0.17465 (5)	0.1010 (3)	
Cl2	0.14916 (9)	0.80981 (5)	0.64259 (4)	0.07445 (19)	
C1	1.4458 (4)	0.6784 (2)	−0.08893 (15)	0.0703 (6)	
C2	1.5551 (4)	0.7730 (2)	−0.07247 (16)	0.0719 (6)	
H2A	1.6975	0.7964	−0.1085	0.086*	
C3	1.4505 (4)	0.83280 (18)	−0.00168 (16)	0.0654 (5)	
H3A	1.5235	0.8969	0.0100	0.079*	
C4	1.2364 (3)	0.79806 (16)	0.05257 (14)	0.0532 (4)	
C5	1.1286 (4)	0.70284 (18)	0.03344 (15)	0.0637 (5)	
H5A	0.9861	0.6788	0.0691	0.076*	
C6	1.2320 (4)	0.6435 (2)	−0.03847 (17)	0.0733 (6)	
H6A	1.1579	0.5808	−0.0525	0.088*	
C7	1.1296 (3)	0.87101 (16)	0.12331 (15)	0.0557 (4)	
C8	0.9716 (3)	0.81969 (14)	0.21872 (14)	0.0487 (4)	
C9	1.1937 (3)	0.63087 (16)	0.31196 (15)	0.0582 (5)	
H9A	1.3439	0.6594	0.3154	0.070*	
C10	0.9086 (4)	0.52367 (17)	0.32519 (19)	0.0736 (6)	
H10A	0.8222	0.4566	0.3425	0.088*	
C11	0.8102 (3)	0.88957 (15)	0.25517 (14)	0.0522 (4)	
H11A	0.7981	0.9669	0.2131	0.063*	
C12	0.6506 (3)	0.86366 (14)	0.35025 (13)	0.0475 (4)	
C13	0.6915 (3)	0.77059 (16)	0.44322 (14)	0.0554 (4)	
H13A	0.8248	0.7190	0.4452	0.067*	
C14	0.5387 (3)	0.75364 (16)	0.53190 (15)	0.0564 (4)	
H14A	0.5684	0.6911	0.5931	0.068*	
C15	0.3405 (3)	0.83027 (15)	0.52953 (14)	0.0524 (4)	
C16	0.2943 (3)	0.92271 (17)	0.43910 (16)	0.0637 (5)	
H16A	0.1595	0.9733	0.4373	0.076*	
C17	0.4495 (3)	0.93960 (15)	0.35136 (15)	0.0586 (5)	
H17A	0.4198	1.0033	0.2911	0.070*	
N1	0.9986 (2)	0.69650 (11)	0.26747 (11)	0.0466 (3)	
N2	0.8100 (3)	0.62670 (13)	0.27539 (14)	0.0621 (4)	
N3	1.1434 (3)	0.52092 (14)	0.34991 (15)	0.0737 (5)	
O1	1.1701 (3)	0.97469 (12)	0.10500 (12)	0.0776 (4)	

### Atomic displacement parameters (Å^2^)

**Table:**

	*U*^11^	*U*^22^	*U*^33^	*U*^12^	*U*^13^	*U*^23^
Cl1	0.1303 (6)	0.1091 (5)	0.0559 (3)	0.0454 (4)	0.0020 (3)	−0.0198 (3)
Cl2	0.0717 (3)	0.0773 (4)	0.0663 (3)	0.0119 (3)	0.0173 (2)	−0.0097 (3)
C1	0.0846 (14)	0.0750 (14)	0.0403 (10)	0.0208 (11)	0.0000 (9)	−0.0016 (9)
C2	0.0633 (12)	0.0815 (15)	0.0578 (12)	0.0082 (11)	0.0113 (9)	0.0014 (10)
C3	0.0629 (11)	0.0632 (12)	0.0620 (12)	−0.0054 (9)	0.0100 (9)	−0.0024 (9)
C4	0.0524 (10)	0.0568 (10)	0.0446 (9)	−0.0020 (8)	0.0029 (7)	−0.0033 (8)
C5	0.0644 (12)	0.0749 (13)	0.0514 (11)	−0.0129 (10)	0.0062 (9)	−0.0143 (9)
C6	0.0929 (16)	0.0733 (13)	0.0536 (11)	−0.0080 (11)	−0.0012 (11)	−0.0158 (10)
C7	0.0547 (10)	0.0540 (11)	0.0542 (10)	−0.0096 (8)	0.0019 (8)	−0.0052 (8)
C8	0.0478 (9)	0.0483 (9)	0.0477 (9)	−0.0061 (7)	−0.0004 (7)	−0.0079 (7)
C9	0.0458 (9)	0.0616 (11)	0.0635 (11)	0.0033 (8)	0.0018 (8)	−0.0117 (9)
C10	0.0772 (14)	0.0486 (11)	0.0915 (16)	−0.0111 (10)	0.0164 (12)	−0.0116 (10)
C11	0.0581 (10)	0.0459 (9)	0.0495 (9)	−0.0011 (8)	−0.0021 (8)	−0.0070 (7)
C12	0.0507 (9)	0.0439 (9)	0.0483 (9)	−0.0003 (7)	−0.0032 (7)	−0.0131 (7)
C13	0.0527 (10)	0.0569 (10)	0.0523 (10)	0.0133 (8)	−0.0012 (8)	−0.0102 (8)
C14	0.0606 (11)	0.0529 (10)	0.0495 (10)	0.0077 (8)	−0.0020 (8)	−0.0051 (8)
C15	0.0532 (10)	0.0534 (10)	0.0514 (10)	0.0010 (8)	0.0013 (8)	−0.0161 (8)
C16	0.0636 (11)	0.0584 (11)	0.0634 (12)	0.0193 (9)	0.0022 (9)	−0.0113 (9)
C17	0.0719 (12)	0.0465 (9)	0.0517 (10)	0.0112 (8)	−0.0022 (9)	−0.0061 (8)
N1	0.0400 (7)	0.0463 (7)	0.0520 (8)	−0.0052 (6)	0.0049 (6)	−0.0101 (6)
N2	0.0496 (8)	0.0545 (9)	0.0815 (11)	−0.0141 (7)	0.0071 (8)	−0.0146 (8)
N3	0.0744 (11)	0.0538 (10)	0.0842 (12)	0.0101 (8)	0.0069 (9)	−0.0065 (8)
O1	0.0930 (10)	0.0584 (9)	0.0789 (10)	−0.0199 (7)	0.0262 (8)	−0.0121 (7)

### Geometric parameters (Å, º)

**Table:**

Cl1—C1	1.745 (2)	C9—N1	1.342 (2)
Cl2—C15	1.743 (2)	C9—H9A	0.9300
C1—C2	1.372 (3)	C10—N2	1.311 (3)
C1—C6	1.379 (3)	C10—N3	1.351 (3)
C2—C3	1.381 (3)	C10—H10A	0.9300
C2—H2A	0.9300	C11—C12	1.461 (2)
C3—C4	1.398 (3)	C11—H11A	0.9300
C3—H3A	0.9300	C12—C13	1.397 (2)
C4—C5	1.389 (3)	C12—C17	1.398 (2)
C4—C7	1.492 (3)	C13—C14	1.377 (2)
C5—C6	1.386 (3)	C13—H13A	0.9300
C5—H5A	0.9300	C14—C15	1.384 (2)
C6—H6A	0.9300	C14—H14A	0.9300
C7—O1	1.224 (2)	C15—C16	1.377 (3)
C7—C8	1.499 (2)	C16—C17	1.375 (3)
C8—C11	1.343 (2)	C16—H16A	0.9300
C8—N1	1.428 (2)	C17—H17A	0.9300
C9—N3	1.311 (3)	N1—N2	1.366 (2)
			
C2—C1—C6	121.6 (2)	N2—C10—H10A	122.1
C2—C1—Cl1	118.80 (18)	N3—C10—H10A	122.1
C6—C1—Cl1	119.6 (2)	C8—C11—C12	130.38 (16)
C1—C2—C3	119.1 (2)	C8—C11—H11A	114.8
C1—C2—H2A	120.5	C12—C11—H11A	114.8
C3—C2—H2A	120.5	C13—C12—C17	117.31 (16)
C2—C3—C4	120.7 (2)	C13—C12—C11	124.22 (15)
C2—C3—H3A	119.6	C17—C12—C11	118.39 (15)
C4—C3—H3A	119.6	C14—C13—C12	121.34 (16)
C5—C4—C3	118.93 (19)	C14—C13—H13A	119.3
C5—C4—C7	123.74 (16)	C12—C13—H13A	119.3
C3—C4—C7	117.23 (18)	C13—C14—C15	119.61 (16)
C6—C5—C4	120.40 (19)	C13—C14—H14A	120.2
C6—C5—H5A	119.8	C15—C14—H14A	120.2
C4—C5—H5A	119.8	C16—C15—C14	120.58 (17)
C1—C6—C5	119.2 (2)	C16—C15—Cl2	119.85 (14)
C1—C6—H6A	120.4	C14—C15—Cl2	119.56 (14)
C5—C6—H6A	120.4	C17—C16—C15	119.36 (17)
O1—C7—C4	120.59 (16)	C17—C16—H16A	120.3
O1—C7—C8	118.20 (17)	C15—C16—H16A	120.3
C4—C7—C8	121.21 (16)	C16—C17—C12	121.78 (17)
C11—C8—N1	122.27 (15)	C16—C17—H17A	119.1
C11—C8—C7	119.71 (16)	C12—C17—H17A	119.1
N1—C8—C7	118.02 (14)	C9—N1—N2	109.33 (15)
N3—C9—N1	110.62 (17)	C9—N1—C8	129.36 (14)
N3—C9—H9A	124.7	N2—N1—C8	121.31 (13)
N1—C9—H9A	124.7	C10—N2—N1	101.71 (16)
N2—C10—N3	115.89 (18)	C9—N3—C10	102.45 (16)
			
C6—C1—C2—C3	1.4 (3)	C17—C12—C13—C14	0.6 (3)
Cl1—C1—C2—C3	−177.75 (14)	C11—C12—C13—C14	177.44 (17)
C1—C2—C3—C4	0.0 (3)	C12—C13—C14—C15	−0.2 (3)
C2—C3—C4—C5	−0.6 (3)	C13—C14—C15—C16	0.4 (3)
C2—C3—C4—C7	−177.11 (17)	C13—C14—C15—Cl2	−179.19 (14)
C3—C4—C5—C6	−0.1 (3)	C14—C15—C16—C17	−1.1 (3)
C7—C4—C5—C6	176.17 (18)	Cl2—C15—C16—C17	178.52 (15)
C2—C1—C6—C5	−2.1 (3)	C15—C16—C17—C12	1.6 (3)
Cl1—C1—C6—C5	177.06 (15)	C13—C12—C17—C16	−1.3 (3)
C4—C5—C6—C1	1.4 (3)	C11—C12—C17—C16	−178.32 (18)
C5—C4—C7—O1	−150.2 (2)	N3—C9—N1—N2	−0.5 (2)
C3—C4—C7—O1	26.1 (3)	N3—C9—N1—C8	178.82 (17)
C5—C4—C7—C8	30.1 (3)	C11—C8—N1—C9	−122.4 (2)
C3—C4—C7—C8	−153.57 (17)	C7—C8—N1—C9	58.0 (2)
O1—C7—C8—C11	25.1 (3)	C11—C8—N1—N2	56.9 (2)
C4—C7—C8—C11	−155.23 (17)	C7—C8—N1—N2	−122.75 (17)
O1—C7—C8—N1	−155.21 (17)	N3—C10—N2—N1	0.0 (2)
C4—C7—C8—N1	24.5 (2)	C9—N1—N2—C10	0.3 (2)
N1—C8—C11—C12	5.7 (3)	C8—N1—N2—C10	−179.08 (16)
C7—C8—C11—C12	−174.66 (17)	N1—C9—N3—C10	0.5 (2)
C8—C11—C12—C13	23.4 (3)	N2—C10—N3—C9	−0.3 (3)
C8—C11—C12—C17	−159.82 (19)		
